# Pomegranate seed hydroalcoholic extract improves memory deficits in ovariectomized rats with permanent cerebral hypoperfusion /ischemia

**Published:** 2015

**Authors:** Alireza Sarkaki, Yaghoub Farbood, Shieda Hashemi, Maryam Rafiei Rad

**Affiliations:** 1 *Dept. of Physiology, Medicine Faculty, Ahvaz Jundishpur University of Medical Sciences, Ahvaz-Iran*; 2 *Physiology Research Center, Ahvaz Jundishpur University of Medical Sciences, Ahvaz-Iran. *; 3 *Dept. of Biology, faculty of Sciences, Islamic Azad University, Izeh branch, Izeh-Iran. *

**Keywords:** *Ovariectomy*, *Cerebralischemia*, *Pomegranate*, *Memory*, *Rat*

## Abstract

**Objectives:** Estrogen deficit following menopause results in cognitive behaviors impairment. This study aimed to evaluate the effects of pomegranate seed extract (PGSE) on avoidance memories after permanent bilateral common carotid arteries occlusion (2CCAO) in ovariectomized (OVX) rats.

**Materials and Methods:** Adult female Wistar rats were divided randomly into eight groups with 8 rats in each group: 1) Sham-operated for ovaries and 2CCAO (ShO); 2) OVX and sham operated for ischemia (OShI); 3-7) OVX with 2CCAO (OI) received PGSE (100, 200, 400 and 800 mg/2ml/kg or normal saline, orally) for 14 days (OI+E100, 200, 400, 800 or OI+Veh); 8) OShI received most effective dose of PGSE (200 and 400 mg/kg for passive and active avoidance memories respectively). Active and passive avoidance tasks were measured in Y-maze and two-way shuttle box respectively. Data were analyzed with one-way and RM-ANOVA followed by HSD post-hoc test.

**Results:** Sensorimotor impaired in OShI+Veh and OI+Veh (P<0.001 vs. ShO). PGSE improved it significantly in dose dependently manner (P<0.001 vs. OI+Veh). Both types of memories were significantly impaired in OVX rats before and after 2CCAO (P<0.001). PGSE treatment significantly improved memories in OI groups (P<0.05, P<0.01 and P<0.001) compared with OI+Veh. No toxicity was observed with PGSE consumption (800 mg/kg, 2 weeks, orally)**. **

**Conclusion:** PGSE exhibits therapeutic potential for avoidance memories, which is most likely related at least in part to its phytoestrogenic and also antioxidative actions.

## Introduction

The effects of combined gonadal hormones deprivation and cerebral hypoperfusion/ischemia on memory is unknown. Therefore, we hypothesized that phytoestrogenic natural substances such as pomegranate seed extract (PGSE) serves as a neurotrophomodulatory substance for some brain areas neurons thought to be involved in learning and memory. 

Gonadal hormones, such as estrogen, can alter cognitive performance. Estrogen can have positive mnemonic effects in the inhibitory avoidance task (Rhodes & Frye, 2004 ▶). Ovarian hormones differently affect female memory in an age-dependent manner (Aguiar et al., 2006 ▶). Surgically menopausal women and estrogen deficits in rats (ovariectomy) impair memory and cognition incur a 2- to 5-fold increased risk for dementia and mortality from neurological diseases, but the mechanisms underlying these increased risks remain unclear (Ben et al., 2010 ▶; Scott et al., 2013 ▶). 

 Estrogen deficit is associated with mental health disorders, emotional difficulties, memory impairment, and other cognitive failures (Sarkaki et al., 2008 ▶). Recently, considerable attention has been paid to medicinal plants and fruits with various physiological activities such as pomegranate and soy as phytoestrogens to alleviate symptoms related to estrogen deficit (Varadinova et al., 2009 ▶). Phytoestrogens are naturally occurring plant-derived compounds that are present in the human diet and are considered selective estrogen receptor (ER) modulators. The phytoestrogens are potent isoflavonoid, with binding affinities for both ER-alpha and ER-beta that are comparable to those of 17 b-estradiol (Canal Castro et al., 2012 ▶).

Stroke is a major cause of adult-onset disability and dependency (Sheorajpanday et al., 2011 ▶). Neuropsychological impairment after stroke when no motor sensory or language deficits are left remains understudied (Planton et al., 2012 ▶). The outcome for menopause women with hypoxic-ischemic brain injury (HIBI) is often poor. It is important to establish an accurate prognosis as soon as possible after the insult to guide management (Howard et al., 2012 ▶). Cerebral ischemia resulted from low oxygen and glucose supply evidently decreases the formation of ATP (Aviram et al., 2002 ▶; Aquilano et al., 2008 ▶). Damage to brain tissue resulting from cerebral ischemia is a major cause of adult disability. It can lead to cognition problems, seizures and death (Aviram et al., 2000 ▶; Aviram & Dornfeld, 2001 ▶). Transient global cerebral ischaemia (forebrain ischaemia), occurring during cardiorespiratory arrest in patients or in experimental animals, induces selective and delayed neuronal cell death (Ben Nasr et al., 1996 ▶; Block, 1999 ▶; Aviram et al., 2000 ▶; Banerjee et al., 2003 ▶). The hippocampus plays a central role in the brain network that is essential for memory function. Paradoxically, the hippocampus is also the brain structure that is most sensitive to hypoxic-ischemic episodes (Lavenex et al., 2011 ▶). Pyramidal neurons in the CA1 region of the hippocampus are particularly vulnerable and die after global ischemia. Hippocampal CA1 sector injury is observed a few days after untreated forebrain ischemia in the rat (Block, 1999 ▶; Aviram et al., 2000 ▶; Aviram & Dornfeld, 2001 ▶; Aviram et al., 2002 ▶; Banerjee et al., 2003 ▶), gerbil and human (Aviram et al., 2000 ▶; Cechetti et al., 2010 ▶). Transient global ischemia induces selective, delayed neuronal death of pyramidal neurons in the hippocampal CA1. Whereas long term treatment of middle-aged female rats with estradiol at physiological doses ameliorates neuronal death, the signaling pathways that mediate the neuroprotection are, as yet, unknown (De Butte-Smith et al., 2012 ▶).

Extracts from natural substances have the ability to protect neurons from ischemic damage (Meyer et al., 1987 ▶; McCarty, 2000 ▶). The extracts have several functions including antioxidant effects in neuroprotection from ischemic insults (Evans, 1993 ▶). Polyphenols have been found to possess antioxidant properties as well as having effects on gene expression (Ito et al., 1975 ▶). Recent studies indicate that among foods which contain polyphenols. Recent studies indicate that among foods that contain polyphenols, juice extracted from the pomegranate has the highest concentration of measurable polyphenols (Takeda et al., 2005 ▶; Jee et al., 2008 ▶). The pharmacologic actions of pomegranate juice include antiatherosclerotic, antibacterial, and antiproliferative properties (Kaplan et al., 2001 ▶; Kim et al., 2002 ▶). Studies of dietary supplementation with pomegranate juice have shown protective effects against atherogenesis and atherosclerosis as well as reductions in serum angiotensin-converting enzyme activity with subsequent reductions in systolic blood pressure (32–34). Also, maternal dietary supplementation with pomegranate juice results in protection against neonatal brain injury (Lau et al., 2005 ▶). 

During recent years, phytoestrogens have been receiving an increasing amount of interest, as several lines of evidence suggest a possible role in preventing a range of diseases. The presence of these phytoestrogenic compounds in pomegranate has been shown to exert suppressive effects on disease (van Elswijk et al., 2004 ▶). Another study via biochemical analysis revealed that pomegranate with highest antioxidant capacity was found in leaves followed by peel, pulp, and seed (O'Keefe & Conway, 1978 ▶). In our previous work (Sarkaki & Rezaiei, 2013 ▶) the beneficial effects of PGSE on adult male and female rats suffering with cerebral hypoxia-ischemia (HI) and Parkinson’s disease were determined (Sarkaki et al., 2013b ▶). 

With regard to the several beneficial effects mentioned for pomegranate, it seems that administration of its seed extract (PGSE) can be effective for the improvement of post-ischemic injuries. On the other hand, female sex hormones such as estrogen are neuroprotective and PGSE also contains phytoestrogenic substances with antioxidative properties, we decided therefore to test the effect of PGSE on cognition deficiency induced by HI in adult female rats. 

The present study investigated the effects of different doses of PGSE on passive and active avoidance memories following cerebral hypoxia-ischemia induced via bilateral common carotid artery occlusion. 

## Materials and Methods

**Animals**

Eighty adult male albino rats of Wistar strain (250±20g, 3-4 months) obtained from Ahvaz Jundishapur University of Medical Sciences (AJUMS) laboratory animal center were used in this study. Animals were housed in standard cages under controlled room temperature (20±2 ◦C), humidity (55-60%) and light exposure conditions 12:12 h light–dark cycle (lighted on 07:00 am). All experiments carried out during the light phase of the cycle (8:00 am to 6:00 pm). Access to food and water were *ad libitum* except during the experiments. Animal handling and experimental procedures performed under observance of the University and Institutional legislation, controlled by the Local Ethics Committee for the Purpose of Control and Supervision of Experiments on Laboratory Animals. All efforts were made to minimize animal suffering, to reduce the number of animals used. Prior to the onset of behavioral testing, all rats were gentle handled for 3 days (daily 10 min). After one week, animals of seven groups were ovariectomized (O) under anesthesia induced by injection of ketamine hydrochloride (90 mg/kg, i.p., Rotex Medica, Trittau, Germany) and Xylazine (10 mg/kg, i.p., Miles Laboratories, Shawnee, Kansas, USA). While ovaries remained intact in sham operated groups (ShO). Bilateral common carotid arteries were occluded and dissected (2CCAO) in ovariectomized rats (OI groups). Animals were divided randomly into eight groups with 8 in each: 1) sham-operated for OVX (ShO); 2) ovariectomized and sham operated for ischemia with manipulation of both common carotids arteries without occlusion received vehicle (OShI+Veh); 3-7) ovariectomized with 2CCAO (OI) received PGSE (100, 200, 400 and 800 mg/2ml/kg or normal saline as vehicle, orally) for 14 days (OI+E100, 200, 400, 800 or OI+Veh); 8) OShI received most effective dose of PGSE which was different in two cognition tests (200 mg/kg, OShI+E200 in shuttle box test and 400 mg/kg, OShI+E400 in Y-maze test). 

**2CCAO procedure**

 Cechetti’s method (2012) with little modification was used. Briefly, rats were anesthetized with ketamine/xylazine (50/5mg/kg, i.p). A neck ventral midline incision was made and the common carotid arteries were then exposed and gently separated from the vagus nerve. Carotids were occluded with three days interval between interventions, the right common carotid being the first to be assessed and the left one being occluded three days later. Sham-operated rats were under same surgical procedures without carotid artery ligation and occlusion (Cechetti et al., 2012 ▶).

**PGSE preparation**

 Pomegranate fruits (*Punica granatum L*.) as large fruit with red barriers were purchased from Shahreza granatum gardens- Iran. Seeds removed from the fruits, air dried in shade for one week and milled to fine powder (electric mill, Panasonic Co. Japan). The seeds powder was macerated in 70% ethanol for 72 hours at room temperature. The ethanol extract evaporated (Rotary Ovaporator, Heidolph Co. Germany) to remove ethanol and PGSE was obtained as a lyophilized powder (yield 17±2%). 

**Treatment**

 Different doses of extract were administrated to each animal in separate groups via forced oral administration (gavage) everyday 8:00–9:00 am for 2 weeks, starting on the 5 days post ischemic injury. Sham treated animals (OI+Veh) were received same volume of normal saline (2ml) for same period. 

**Sensorimotor evaluation**

 It consisted of two tests developed and described by Garcia (Garcia et al., 1995 ▶) with some modifications as discussed below. The scores assigned to each rat at the end of each examination is the sum of the two test scores. The minimum neurological score is three and the maximum is four. I/R rats with at least score 3 in each test were selected in this study. 

**Spontaneous activity**

 The animal is observed for 5 min in its normal cage. Scores indicate the following: (1) rat moves around, explores the environment, and approaches at least three walls of the cage; (2) rat moves around in the cage but does not approach all sides and hesitates to move, although it eventually reaches at least one upper rim of the cage (height=10 cm); (3) rat dose not rise up at all and barely moves in the cage; (4) rat does not move at all. 

**Symmetry in the movement of four limbs**

The rat is held in the air by the tail to observe symmetry in the movement of the four limbs. Scores indicate the following: (1) all four limbs extended symmetrically; (2) limbs on one side extended less or more slowly than those on the other side; or slow extension of the four limbs; (3) limbs on one or both sides show minimal movements; (4) forelimbs on one or both sides do not move at all.

**Passive avoidance task**

 The apparatus used for evaluation the passive avoidance task was two-way shuttle box (Borj Sanaat Co. Tehran, Iran), which consisted of two adjacent plexiglas compartments of identical dimensions (27×14.5×14 cm) with grid floors. The floor of two compartments has been covered with stainless steel bars (2 mm diameter) with 1 cm distance. Light compartment was illuminated by a 5 W lamp mounted on its wall just below a movable transparent plexiglas ceiling. The Tamburella’s procedure with little modification was used for passive avoidance memory test (Tamburella et al., 2012 ▶). Briefly, each rat was allowed a 10 minutes adaptation period with free access to either the light or dark compartment of the box to avoidance training and after being placed in a shuttle-box (in order to familiar with instruments). Following this adaptation period, on the second day (initial phase) rats were placed into the illuminated compartment and 10 seconds later the sliding door was raised. Initial latency was recorded as learning phase. Upon entering the dark compartment the door was closed and a 1.5 mA constant-current was applied to animal fore and hind paws for 3 seconds as electrical shock. After 60 seconds (in order to consolidation and return to normal psychological state) the rat was removed from the dark compartment and placed into the home cage. In order to test short-term memory, 24 hours after receiving foot shock, the rats were placed in illuminated chamber again and the sliding door was raised at 30 seconds later. The latency of entering the dark compartment was recorded again as memory test (step-through latency). The maximum time that considered in this procedure were 300 seconds (Mahut et al., 1982 ▶; Levy et al., 1985 ▶; Lipton & Rosenberg, 1994 ▶).

**Active avoidance task**

 The apparatus used for evaluation the active avoidance task was 3-equal arms Y-maze (Made in Ahvaz, Iran). The Yu and Besnard procedures with little modifications were used for active avoidance memory test (Besnard et al., 2012 ▶; Yu et al., 2012 ▶). Sham operated, ischemic and all treated rats were trained in equal 3-arms Y-maze with using an A/D converter, special software on a PC as active avoidance learning. Training was done, 30 trails daily for 4 days as four sessions. Animals were conditioned, using a 12 watts light as conditioned stimulus (CS) and 20-25 volts, 3 mA electrical foot shock as unconditioned stimulus (UCS). Inter-trials interval (ITI) and inter-stimuli interval (ISI) were 60 and 5 seconds, respectively. Trained animals left the dark arms and enter in light arm during 5 seconds delay time (ISI). This effort was counted as conditioned response. Criterion condition response (CCR) was 90 percents correct responses fearing from unsafe locations in Y-maze (darken arms) to lighted (safe arm) in last session of training as optimum level of learning. Training session’s number was same for all rats in different groups.

**Statistical analysis**

 Data were expressed as mean ±SEM of values for sensorimotor and avoidance memories tests. Statistical analysis was performed by one-way ANOVA (for sensorimotor and passive avoidance task) and repeated measures (RM) ANOVA (for active avoidance task) followed by HSD post hoc test. A P-value less than 0.05 were assumed to denote a significant difference and levels of significance are indicated by symbols: * vs. ShO group and # vs. OShI+Veh or OI+Veh groups. 

## Results

**Motor evaluation**

**Figure 1:**
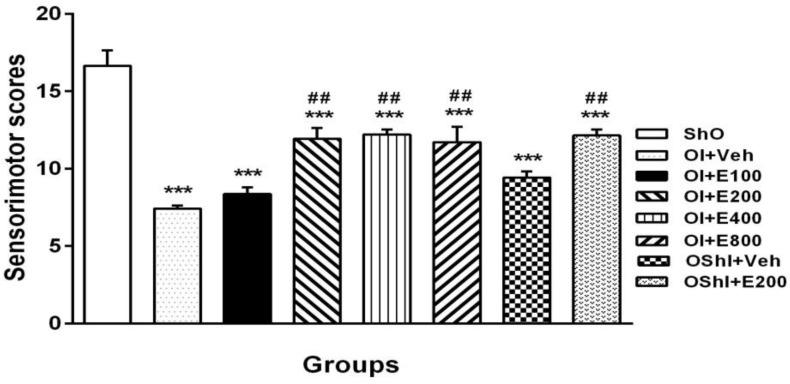
Mean±SEM of sensorimotor scores in different groups 5 days after 2CCAO induction. ***P<0.001 for significant difference with ShO group and ##P<0.01 for significant difference versus OI+Veh groups. One-way ANOVA analysis that followed by HSD post hoc test. ShO: sham operated for ovariectomy, OI: ovariectomized rats with 2CCAO, OI+E100-800: ovariectomized rats with 2CCAO received different doses of PGSE, OI+Veh: ovariectomized rats with 2CCAO received normal saline, OShI+E200 and OShI+Veh: ovariectomized rats with sham operated for 2CCAO received 200 mg/kg PGSE and normal saline respectively.

Figure 1 shows the sensorimotor scores 5 days after 2CCAO operation. There was significant sensorimotor impairment in OShI+Veh and OI+Veh groups when compared to ShO group (P<0.001). 

Treatment with different doses of PGSE for two weeks improved sensorimotor scores significantly in OI+E200, OI+E400 and OI+E 800 groups, but not with dose 100 mg/kg (P<0.001 vs. OI+Veh), while there was not any difference between them. There were significant differences between OShI+Veh vs. ShO (P<0.001) and OShI+E200 vs. OShI+Veh groups respectively.

**Passive avoidance memory**

**Figure 2:**
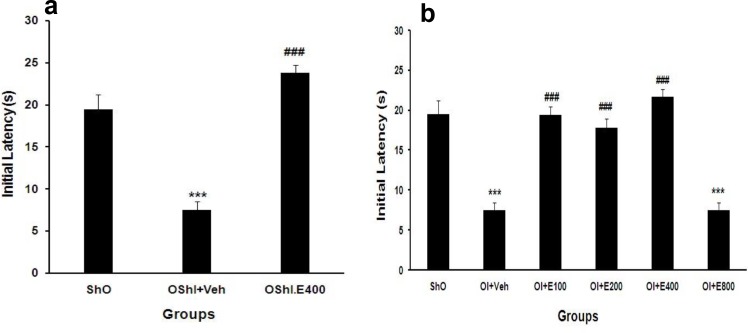
Mean±SEM of initial latency in different groups after two weeks treatment with PGSE in ovariectomized without and with 2CCAO groups. ***P<0.001 for significant difference with ShO group and ###P<0.001 for significant difference versus OI+Veh groups. One-way ANOVA analysis that followed by HSD post hoc test. ShO: sham operated for ovariectomy, OI: ovariectomized rats with 2CCAO, OI+E100-800: ovariectomized rats with 2CCAO received different doses of PGSE, OI+Veh: ovariectomized rats with 2CCAO received normal saline, OShI+E200 and OShI+Veh: ovariectomized rats with sham operated for 2CCAO received 200 mg/kg PGSE and normal saline respectively.

As shown in figure 2 the initial latency for leaving rats from lighted to darken compartment of shuttle box before exposing them to any serious stimulus (electrical shock) in ovariectomized rats without 2CCAO that received normal saline (OShI+Veh) was decreased significantly (P<0.001) when compared with sham operated (ShO) group. In this test, 200 mg/kg of PGSE was the best effective dose in passive avoidance memory and treatment OShI group with this dose (OShI+E200) reversed it toward to normal range (P<0.001, figure 2a).

Treatment OVX animals without ischemia with this effective dose of PGSE (OShI+E200) compensated it and reversed its level in ShO group (fig.2a). Treatment of OI rats with oral administration of 100, 200 and 400 mg/kg of PGSE for 14 days improved initial latency significantly when compared with OI+Veh group (P<0.001), while initial latency of OI+E800 was reversed significantly when compared with ShO as well as other treated groups with PGSE (P<0.001, fig. 2b). Short-term passive avoidance memory (24 hours after exposing the electrical shock to paws of rats) in all groups was evaluated with same procedure while no shock delivered to animals. 

**Figure 3:**
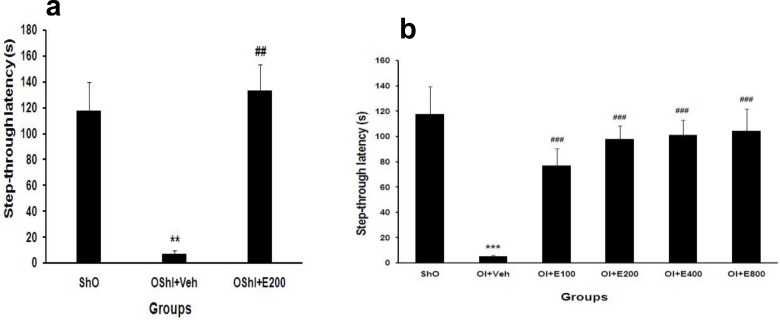
Mean±SEM of step-through latency in different groups after two weeks treatment with PGSE in ovariectomized without and with 2CCAO groups. ***P<0.001 for significant difference with ShO group and ###P<0.001 for significant difference versus OI+Veh groups. One-way ANOVA analysis that followed by HSD post hoc test. ShO: sham operated for ovariectomy, OI: ovariectomized rats with 2CCAO, OI+E100-800: ovariectomized rats with 2CCAO received different doses of PGSE, OI+Veh: ovariectomized rats with 2CCAO received normal saline, OShI+E200 and OShI+Veh: ovariectomized rats with sham operated for 2CCAO received 200 mg/kg PGSE and normal saline respectively.

As shown in figure 3 step-through latency (memory) was impaired significantly in both OShI+Veh and OI+Veh groups (P<0.01 and P<0.001 vs. ShO respectively, fig.3a). Treatment of ovariectomized animals without ischemia with best effective dose of PGSE (200 mg/kg) and ovariectomized animals with ischemia with different doses of extract improved the memory significantly when compared with OI+Veh rats (P<0.001, fig. 3b). 

**Active avoidance memory**

**Figure 4:**
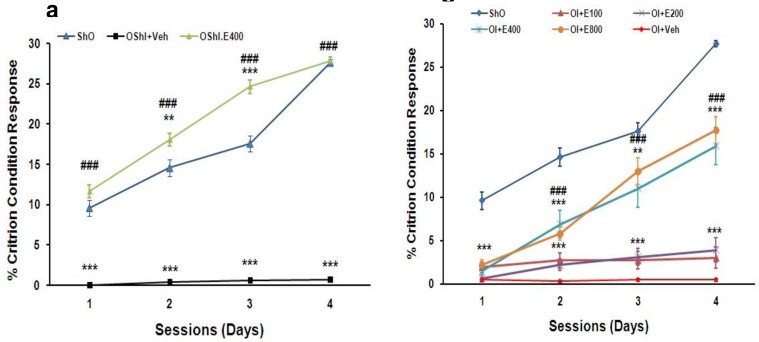
The percents of criterion condition responses (%CCRs) during four sessions training in Y-maze in different groups after two weeks treatment with PGSE in ovariectomized without and with 2CCAO groups. ***P<0.001 for significant difference with ShO group and ###P<0.001 for significant difference versus OShI+Veh and OI+Veh groups. Repeated measured (RM) ANOVA analysis that followed by HSD post hoc test. ShO: sham operated for ovariectomy, OI: ovariectomized rats with 2CCAO, OI+E100-800: ovariectomized rats with 2CCAO received different doses of PGSE, OI+Veh: ovariectomized rats with 2CCAO received normal saline, OShI+E200 and OShI+Veh: ovariectomized rats with sham operated for 2CCAO received 200 mg/kg PGSE and normal saline respectively.

Figure 4 shows that the percents of criterion condition responses (%CCRs) was lower significantly after ovariectomy without 2CCAO group (OShI+Veh) during 4 days training in Y-maze (P<0.001). In this test, 400 mg/kg of PGSE was the best effective dose in active avoidance memory and treatment OShI group with this dose (OShI+E400) reversed it to normal range (P<0.001, fig. 4a).

Results of treatment the ovariectomized with 2CCAO groups with different doses of PGSE showed that only doses 400 and 800 mg/kg could improve %CCRs during 2-4 sessions significantly (P<0.001), with no significant difference. But it is steel lower than ShO group significantly during those training sessions (P<0.01 and P<0.001, fig. 4b).

## Discussion

Our results show that passive and active avoidance tasks impair after ovarectomy in rats because of removing peripheral source of estrogen and treatment them with PGSE as a natural product with phytoestrogenic content agent could reverse avoidance tasks significantly. On the other hand, cerebral hypoperfusion induction in ovariectomized rats with 2CCAO impaired both passive and active avoidance tasks significantly while administration of doses 200 mg/kg (for passive avoidance task) and 400 mg/kg (for active avoidance task) had significant improving effects. These results show that 2CCAO induction causes oxidative stress progression and free radicals production in brain tissues and PGSE as an estrogenic compound and a potent antioxidant could activate estrogen beta receptors (Sarkaki et al., 2013a ▶; Sarkaki et al., 2013b ▶; Sarkaki & Rezaiei, 2013 ▶) as well as scavenge free radicals from brain regions involving the cognitive functions.

Ischemic stroke is a devastating condition, for which there is still no effective therapy. Acute ischemic stroke is associated with high concentrations of glutamate in the blood and interstitial brain fluid. The inability of the tissue to retain glutamate within the cells of the brain ultimately provokes neuronal death. Increased concentrations of interstitial glutamate exert further excitotoxic effects on healthy tissue surrounding the infarct zone (Godino Mdel et al., 2013 ▶). 

Ovariectomy is known as 'surgical menopause' with decreased levels of oestrogen in female rodents and its reported risks and adverse effects include cognitive impairment. In the brain, oestrogen exerts effects through its receptors, oestrogen receptor alpha (ERalpha) and beta (ERbeta) (Qu et al., 2013 ▶). We hypothesize that menopause is an aging phenomenon that combines with oxidative stress and free radicals generation. Estrogen deficit following menopause results in cognitive behaviors impairment (Sarkaki et al., 2008 ▶). Phytoestrogens and other plant-derived compounds and extracts have been developed for the treatment of menopause-related complaints and disorders. Since estrogens have been discussed to enhance the risk for hormone-sensitive cancers, research activities try to find alternatives. Phytoestrogens like genistein, ellagic acid, and resveratrol presented in PGSE as well as other plant-derived compounds are capable of substituting for estrogens to some extent (Schilling et al., 2014 ▶). 

Estrogens are not only critical for sexual differentiation; it is well-known for the role of 17beta-estradiol (E2) in the adult brain modulating memory, learning, mood and acts as a neuroprotector. As estrogen levels change with age, especially in females, it is important to know the effects of low E2 levels on ERalpha distribution; results from previous studies are controversial regarding this issue (Navarro et al., 2013 ▶).

Treatments for protection against neuronal cell death induced by hypoxia-ischemia (HI) and reperfusion have been developed in recent years, but none has been highly successful. A fundamental process believed to be responsible for HI damage to neurons is excitotoxicity, triggered mainly by elevated extracellular glutamate concentration (Choi & Rothman, 1990 ▶). This ischemia-induced release of glutamate likely occurs in man as well, and possibly underlies selective damage to the human hippocampus (Chun et al., 2008 ▶). Glutamate may cause ischemic neuronal death by acting at excitatory N-methyl-Daspartate (NMDA) receptors (Colbourne et al., 1999 ▶) which play an important physiological role in memory (Collingridge et al., 1983 ▶). Thus, the high concentration of NMDA excitatory receptors on the dendrite trees of hippocampal CA1 pyramidal cells (Esposito et al., 2002 ▶) likely explains the long-known selective vulnerability of the CA1 zone of the hippocampus to ischemic brain damage. 

Estrogen deprivation after menopause is associated with increased oxidative stress. Oxidative stress and inflammation result in the development of ovariectomy-induced pathophysiological changes (Kaur et al., 2013 ▶). Reactive oxygen species (ROS) are generated within brain tissue during HI and play a role in the development of cerebral damage. They may be directly involved in glutamate release (Floyd, 1999 ▶) and more importantly, they may participate in the excitotoxic process itself. ROS are extremely reactive and attack lipids, proteins, and nucleic acids, which eventuates in tissue injury and cell death (Gil et al., 2000 ▶). Although there is strong evidence that total destruction of hippocampal CA1 neurons is sufficient to cause a memory deficit (Greenamyre et al., 1985 ▶), it is still presently unclear to what degree subtotal ischemic hippocampal damage may occur without an ensuing memory deficit (Hashimoto et al., 2003 ▶). The free radicals are neutralized by an elaborate antioxidant defense system consisting of enzymes and numerous non-enzymatic antioxidants, including vitamins A, E and C, glutathione, ubiquinone, and flavonoids (Dumlu & Gurkan, 2007 ▶; Pilsakova et al., 2010 ▶). 

Hormone therapy to postmenopausal females increases the risk and severity of ischemic stroke. Previous work using an animal model of menopause (reproductive senescence) showed that middle cerebral artery occlusion (MCAO) causes a larger cortical-striatal infarct in this older acyclic group compared with younger females. Moreover, although estrogen treatment is neuroprotective in younger females, estrogen paradoxically increases infarct volume in acyclic females. These data support the hypothesis that stroke severity in older females is associated with decreased IGF-1 and further indicate that short-term post-ischemic IGF-1 therapy may be beneficial for stroke (Selvamani & Sohrabji, 2010 ▶). 

The results of present study shows the PGSE is a natural product with power phytoestrogenic and antioxidative effects without any side effects could improve the cognitive deficit during menopause period and specially as followed by stress oxidative and possible cerebral hypoperfusion induced by vascular obstruction. 

Our results show that passive and active avoidance tasks impair after ovarectomy in rats because of removing peripheral source of estrogen and treated them with PGSE as a natural product with phytoestrogenic and antioxidant contents could reverse both active and passive avoidance tasks significantly in OVX rats suffer with cerebral HI. Our previous results showed that 2CCAO induction causes oxidative stress progression and free radicals production in brain tissues (Mansouri et al., 2013 ▶) and PGSE as a potent phytoestrogenic and antioxidant may have compensating effects for peripheral estrogen deficit and scavenge produced free radicals after HI from brain regions involving the cognitive functions.
